# Copper transporters are responsible for copper isotopic fractionation in eukaryotic cells

**DOI:** 10.1038/srep44533

**Published:** 2017-03-17

**Authors:** Jean-Loup Cadiou, Sylvain Pichat, Victor P. Bondanese, Alexandre Soulard, Toshiyuki Fujii, Francis Albarède, Philippe Oger

**Affiliations:** 1 Univ Lyon, ENS de Lyon, Université Claude Bernard Lyon 1, CNRS, UMR 5276, Lyon, France; 2 Univ Lyon, Université Claude Bernard Lyon 1, INSA-Lyon, CNRS, UMR5240, Villeurbanne, France; 3 Division of Sustainable Energy and Environmental Engineering, Graduate School of Engineering, Osaka University, Osaka, Japan

## Abstract

Copper isotopic composition is altered in cancerous compared to healthy tissues. However, the rationale for this difference is yet unknown. As a model of Cu isotopic fractionation, we monitored Cu uptake in *Saccharomyces cerevisiae*, whose Cu import is similar to human. Wild type cells are enriched in ^63^Cu relative to ^65^Cu. Likewise, ^63^Cu isotope enrichment in cells without high-affinity Cu transporters is of slightly lower magnitude. In cells with compromised Cu reductase activity, however, no isotope fractionation is observed and when Cu is provided solely in reduced form for this strain, copper is enriched in ^63^Cu like in the case of the wild type. Our results demonstrate that Cu isotope fractionation is generated by membrane importers and that its amplitude is modulated by Cu reduction. Based on *ab initio* calculations, we propose that the fractionation may be due to Cu binding with sulfur-rich amino acids: methionine and cysteine. In hepatocellular carcinoma (HCC), lower expression of the *STEAP3* copper reductase and heavy Cu isotope enrichment have been reported for the tumor mass, relative to the surrounding tissue. Our study suggests that copper isotope fractionation observed in HCC could be due to lower reductase activity in the tumor.

As other transition metals, copper (Cu) is an essential micronutrient for living organisms, but can become toxic if in excess. At low concentrations, Cu is essential for the activity of proteins such as cytochrome c oxidase, which is involved in mitochondrial respiration. At high concentrations, Cu generates reactive oxygen species that are highly toxic^1^,^2^. Therefore, living organisms have evolved regulatory mechanisms to control the cellular speciation and content of this metal and avoid its toxicity^3^. Transmembrane proteins allow controlled cellular import and export, and Cu is transported between different organs tightly bound to organic molecules. In contrast, many cancer types exhibit a dysregulation of cellular Cu homeostasis^4^,^5^. Several eukaryotes seem to react similarly to some cancers: Cu concentration in the blood-serum of healthy mice or humans is lower than that of tumor-bearing mice or human patients affected by breast, lung, or colon cancers^5^, respectively. A way of better understanding dysregulation of transmembrane Cu transport is to use Cu isotopes. Indeed, in a preliminary study, Fosset *et al*.^6^ used copper enriched in ^65^Cu for Cu fluxes tracing in cells. Instead natural variability of ^65^Cu/^63^Cu along biochemical pathways, a method similar to that has been used to trace fluxes of various elements through various geological reservoirs^7^, has been used to trace fluxes in living cells or among organs. It has been found that the ^65^Cu/^63^Cu ratio (reported as δ^65^Cu, *i.e.* the relative enrichment of ^65^Cu relative to a reference material: δ^65^Cu = 1000 × [(^65^Cu/^63^Cu)_sample_/(^65^Cu/^63^Cu)_standard_ − 1]) in blood serum decreases with cancer progression^8^. The red blood cells δ^65^Cu values were found to be lower for patients with hepatocellular carcinoma (HCC) relative to healthy subjects, and accompanied by an increase of δ^65^Cu in the tumor mass compared to peritumor tissue^9^. To date, the explanations of these isotopic variations observed between healthy and HCC patients remain unclear^10^. Because these variations involve affinity interactions or changes of valence, several steps of Cu transport and translocation between organs, such as Cu reduction, Cu transport through the membrane or Cu fixation on organic ligands, have the potential to generate fractionation. Interestingly, it has been demonstrated that during the development of some cancers, there is a dysregulation of the expression of Cu importers^11^ and/or of reductases^12^,^13^ in cancerous cells. Here we explore the possibility that the dysregulation of Cu homeostasis is responsible for the modification of the cellular Cu isotopic composition.

To test this hypothesis, we used the yeast model-organism *Saccharomyces cerevisiae*, a long-recognized genetic model for Cu metabolism in eukaryotes, which transmembrane Cu transport mechanisms (Fig. 1) are very similar to those of human cells (review by Nevitt *et al*.^3^), and which is prone to genetic manipulation. In *S. cerevisiae*, Cu is first reduced by reductases such as the Fre1 and Fre2 proteins (*Fre1p* and *Fre2p*)^14^,^15^. Reduced Cu enters the cell through two high-affinity importers Ctr1p^16^ and Ctr3p^17^ as well as through low-affinity transporters such as Fet proteins^18^. The expression of the *FRE1, FRE2, CTR1* and *CTR3* genes is controlled by the *Mac1p* transcription factor^19^. Under low Cu, *Mac1p* induces the expression of both *FRE1* and the high-affinity Cu transporters. Under high Cu conditions, the expression of the *MAC1* gene and the activity of *Mac1p* is inhibited, leading to the lower expression of *CTR1, CTR3*, and *FRE1*^14^,^20^,^21^,^22^. *CTR1* is further regulated at the protein level through endocytosis and vacuolar degradation^23^,^24^,^25^. Here, we have monitored Cu uptake and isotopic composition of *S. cerevisiae* mutants, with deletions of high-affinity transporters or impaired Cu reductase activity, in order to identify and quantify the isotopic fractionation associated which each step of the transport process. Our study demonstrates that copper uptake leads to isotopic fractionation between cells and growth-medium, and that this fractionation is generated by Cu importers and modulated by Cu reductases.

## Materials and Methods

### *Saccharomyces cerevisiae* genotypes and culture conditions

Four strains of *S. cerevisiae* were used in this study: a wild type (DTY165), a Δctr1Δctr3 mutant (MPY17), a Δctr1Δmac1 mutant (SKY34) and a SKY34 strain where *FRE1* was expressed under the constitutive promoter of ADH (alcohol dehydrogenase). See detailed genotypes in Table 1. DTY165, MPY17, and SKY34 strains were kindly provided by the Thiele Laboratory (Duke University Medical Center, USA). *S. cerevisiae* cells were routinely maintained in Yeast-extract Peptone Dextrose (YPD) medium, which contains low concentrations of Cu (0.25 μmol/l). For our experiments, we used the Yeast Nitrogen Base (YNB) growth medium which contains 0.15 μmol/l of Cu.

### Genetic manipulations

Expression of FRE1 was re-established in strain SKY34, by substituting the endogenous *FRE1* promoter (Mac1p dependent) with the ADH promoter. The PCR product obtained using the plasmid pYM-N6 26 and the following primers (forward: CTAATTTCTCGCATATTCACGCCGACGGAAGAACGAGCCGGATCAATATGCGTACGCTGCAGGTCGAC; reverse: ACCGTAGCAAAAAAAGATATAAATAAGCAGAATAATACACGGGTTCTAACCATCGATGAATTCTCTGTCG) was transformed into SKY34 cells followed by selection on YPD-agar plates containing 200 mg/l of G418 (Gibco). Successful promoter substitution was confirmed by PCR screen using the forward primer AACCCAAACATTTTCGCCGA (upstream the recombination region) and the reverse primer KanB1 TGTACGGGCGACAGTCACAT (annealing within the resistance cassette).

### Cell quantification

Cells were quantified by three different approaches. First, cell growth was monitored by measuring the culture optical density (OD) at 600 nm. Second, cells were counted directly using a Thoma cell. Third, in order to normalize the activity of transporters, total cell protein content was measured using a procedure adapted from Zhang *et al*.^27^ and Kushnirov *et al*.^28^.

### Uptake and adsorption experiments

Uptake experiments were started from pre-cultures grown in YPD at 30 °C with 160 rpm shaking and harvested in late exponential phase. Cells were inoculated at a final concentration of 10^8^ cell per ml in fresh 500 ml of YNB containing 2% D-Glucose. The inoculated cultures were doped with 80 μmol/l of Cu (δ^65^Cu = −0.23 ± 0.06‰, 2σ, N = 33) at the start of the uptake experiment (t = 0) and incubated at 25 °C with shaking. At each time point, 1 ml of the culture was sampled for cell quantification and 50 ml for Cu elemental and isotopic analyses. Cells were pelleted by centrifugation at 10000 rpm and 2 °C. Cells were washed once with YNB and centrifuged. The cell pellet was then processed for metal analysis. For each time point, 1 ml of the culture supernatant was sampled to measure the concentration and isotopic composition of the culture medium. To test the impact of the Cu oxidation state on its import, similar uptake experiments were performed in presence of 1 mM of ascorbic acid, a strong reducing agent.

Cu adsorption on *S. cerevisiae* cells was monitored as described previously^29^ using the experimental conditions described above for uptake experiments, except that cells were incubated on ice to inhibit biological activity, *e.g*. copper uptake by cells.

Preliminary experiments conducted during the development of our experimental settings have shown that the Cu concentration in *S. cerevisiae* cells did not vary significantly between ca. 120–180 min and 240 min and that the number of cells stayed stable between 0–240 min. Some cellular division could occur after ca. 260 min hence possibly modifying the Cu concentration in *S. cerevisiae* cells. Thus, to avoid the effect of cell division on Cu concentration, and a potential impact on Cu isotopic composition, the uptake experiments were limited to 0–240 min. The observation of a plateau for Cu concentration reached after 120–180 min, depending on the strain, is in good agreement with previous studies^18^. Two or three independent experiments, hereafter referred to as “replicate experiments”, were performed, with the exception of experiments involving SKY FRE (see Table S1 for details).

### Quantification of cellular Cu reduction activity

Cu reduction was measured using a reductase assay modified from Georgatsou *et al*.^14^,^30^. Briefly, *S. cerevisiae* cells grown as described above were harvested in the late exponential phase. Cells were inoculated at a concentration of 10^9 ^cells/ml of YNB supplemented with 5% D-Glucose, 2 mM bathocuproine disulfonic acid (BCS) - a Cu chelator that becomes red in the presence of Cu(I) -, and 1 mM CuSO_4_, and incubated with shaking at 25 °C for 40 min. Since BCS-Cu complexes have a stoichiometry of 2:1^31^, our assay contains twice as much BCS as Cu. Reduced Cu was quantified by measuring the optical density at 482 nm^14^.

### Chemical separation and purification procedures

Cell samples were dissolved following a procedure adapted from the study of Moynier *et al*. on plants^32^. After weighing, samples were placed in 90 ml PTFE jars and dissolved by sequential addition of concentrated HNO_3_, then a mixture of concentrated HNO_3_:H_2_O_2_ 30% (50:50, v-v). After evaporation, samples were placed in H_2_O_2_ 30% under UV light for two hours at room temperature. The photolysis induced by UV light destroys organic matter^33^, which improves the accuracy of the subsequent metal concentration and isotope analyses.

Cellular Cu was separated and purified by anion-exchange chromatography following the procedure described in Maréchal *et al*.^34^. Briefly, the sample was loaded on 1.6 ml of macroporous anion-exchange resin (AG-MP1, 100–200 mesh, Bio-Rad) in 1 ml of 7 N HCl - H_2_O_2_ 0.001%. The matrix was then eluted in 10 ml 7 N HCl - H_2_O_2_ 0.001%, finally Cu was eluted in 20 ml 7 N HCl - H_2_O_2_ 0.001%. This process was repeated once to ensure a complete purification of Cu from the matrix. The whole procedure blank was 8 ng, which is well below the amount of Cu in *S. cerevisiae* samples (>300 ng).

For the growth medium, the separation procedure had to be adapted because of the high organic content of the medium. Briefly, the first purification step was performed with a resin (1.6 ml of AG-MP1, 100–200 mesh, Bio-Rad) conditioned with 20 ml of a mixture of 80% EtOH (96%): 20% (7 N HCl- H_2_O_2_ 0.001%) (v:v), then the sample was loaded in 1 ml of 7 N HCl-H_2_O_2_ 0.001%. The matrix was eluted in 50 ml of 80% EtOH (96%): 20% (7 N HCl- H_2_O_2_ 0.001%) (v:v) followed by the elution of Cu in 30 ml of 7 N HCl-H_2_O_2_ 0.001%. A second purification step was performed as described above for *S. cerevisiae* samples.

### Elemental and isotopic Cu measurements

Cu concentrations were measured by Quadrupole Inductively-Coupled Plasma Mass-Spectrometry (Q-ICP-MS, Agilent 7500cx) and Cu stable isotope compositions by Multi-Collector Inductively-Coupled Plasma Mass-Spectrometry (MC-ICP-MS, Nu500 HR, Nu Instrument) in wet plasma mode. For this purpose, 50 ml aliquots were taken from the culture medium at *ca*. 10^8 ^cells/ml for each time point (see details in the “Uptake and adsorption experiments” subsection). The instrumental mass fractionation was corrected for using Zn-doping and standard-sample bracketing following the procedure developed by Maréchal *et al*.^34^. Samples were diluted to match the concentration of the standard mixture (typically Zn 0.3 ppm - Cu 0.3 ppm). The mass-dependence of isotope fractionation has been systematically checked. The δ^65^Cu is reported relative to the isotopic solution reference material NIST SRM 976. Measurements were repeated several times over the course of independent MC-ICP-MS sessions (see Supplementary Table S1 for details), hereafter referred to as “replicate measurements”. The long-term external reproducibility was assessed by measuring (1) aliquots of the same *S. cerevisiae* sample (Supplementary Table S2) in four runs over the course of three months: δ^65^Cu = −1.47 ± 0.04‰ (2σ, N = 8), and (2) the Cu solution used for *S. cerevisiae* cultures doping, which was analyzed for each batch of cultures: δ^65^Cu = −0.23 ± 0.06‰ (2σ, N = 33). Thus, we define the external error for our measurements at 0.06‰ (2σ).

## Results

### Effect of Cu uptake on Cu isotopes fractionation by *S. cerevisiae* cells

During uptake experiments, the Cu concentration in the wild type strain (DTY165) and the high-affinity importer knockout strain (MPY17) increased quickly and reached a plateau after approximately 120 minutes (Fig. 2). It is worth noting that a Cu concentration plateau reached after *ca*. 120 min is in agreement with previous studies conducted on Cu uptake by *S. cerevisiae*^18^. It can be considered that this time signals the compensation of Cu import by Cu export. We found that the plateau is remarkably high, reaching 16.6 pmol per 10^6^ cells, in MPY17 - the strain lacking high-affinity importers – relative to DTY165 (9.7 pmol per 10^6^ cells) (Fig. 2).

The contribution of Cu adsorption on Cu content in *S. cerevisiae* cells was evaluated by uptake experiments on ice, which limits active import. Our results show that adsorption on *S. cerevisiae* cells is very limited and does not vary with time (Fig. 3) and therefore does not contribute significantly to the cellular Cu content.

The experimental conditions were designed so that Cu uptake by cells was negligible relative to the metal stock in the growth medium. Concentration in the medium throughout the experiments remained indeed stable, within analytical error ( ± 5%), around the initial concentration (Supplementary Fig. S1). We also confirmed that Cu uptake by cells did not impact the isotopic composition of the medium (Supplementary Fig. S1). The Cu stock in the growth medium can therefore be considered infinite relative to *S. cerevisiae* uptake. Its isotopic composition (δ^65^Cu_medium_) at any given time is equal to that of the Cu doping solution (δ^65^Cu = −0.23‰ ± 0.06‰, 2σ, N = 33). The cellular isotopic composition relative to the growth medium at time t can be defined as Δ^65^Cu (t) = δ^65^Cu_cell_ (t) − δ^65^Cu_medium_. To compare the evolution of cellular isotopic compositions over time between the various strains, we subtracted the initial Δ^65^Cu (0) value for each strain to the Δ^65^Cu (t) at each sampling time t. Thus, we define Δ^65^Cu_cell_ (t) = Δ^65^Cu (t) − Δ^65^Cu (0) = δ^65^Cu_cell_ (t) − δ^65^Cu_cell_ (0). Finally, each value was normalized to 10^8^ cells and is thus expressed as ‰/10^8^ cells. An example of the full calculation is given in Table S1.

The Δ^65^Cu_cell_ decreases with time and reaches a plateau at −0.85 ± 0.06‰/10^8^cells for DTY165 and −0.67 ± 0.30‰/10^8^cells for MPY17 (Fig. 2). Therefore, when the plateau is reached, the presence or absence of copper high-affinity transporters does not seem to have a major influence on Cu fractionation in *S. cerevisiae*.

### Impact of Cu oxidation state on *S. cerevisiae*^65^Cu/^63^Cu

In solution, at neutral pH, Cu is present as Cu(II), whereas transport through the membrane involves Cu(I)^15^. Before entering cells, Cu is reduced by two *S. cerevisiae* proteins: *Fre1p* and *Fre2p*^14^,^15^. The impact of the copper oxidation state on fractionation was addressed through three different approaches. We first tested the evolution of Cu uptake and isotopic composition of a *MAC1* knockout *S. cerevisiae* strain (SKY34). Such mutation leads to a lower Cu reduction activity compared to the wild type. Indeed, *Mac1p* allows *FRE1* transcription by binding to its promoter region, thus in a *MAC1* knockout the expression of *FRE1* is largely suppressed^14^. It was verified that the overall Cu reduction ability of SKY34 is greatly impaired, being three times lower (1.25 ± 0.003 μmol.l^−1^.min^−1^) than in the wild type strain DTY165 (3.62 ± 0.12 μmol.l^−1^.min^−1^) (Fig. 4). Our analyses show that the Cu content measured in SKY34 is not significantly different from that measured in the wild type DTY165 (Fig. 5). Although the *MAC1* knockout and the lower Cu reduction ability of the SKY34 strain show no effect on Cu uptake, it affects the isotopic composition relative to the wild type or MPY17 cells with deleted high-affinity importers. Indeed, the Δ^65^Cu_SKY34_ values oscillated around −0.01 ± 0.08‰/10^8^cells (2σ, N = 10) between t = 0 and 240 min (Fig. 5). One possible explanation for this observation is the low availability of Cu(I) for transport.

To test whether Cu reduction is the limiting step for Cu uptake and monitor the effect on Cu isotopic fractionation, we cultured SKY34 cells in a growth medium containing exclusively Cu(I). In effect, the addition of ascorbic acid to the growth-medium reduces copper in solution to its monovalent form (see the Experimental Procedures section for details). Copper uptake by SKY34 cells under these conditions was increased by a factor of six and the Δ^65^Cu_cell_ of SKY34 cells reached plateau values (−0.90 ± 0.02‰/10^8^ cells) comparable to those of the DTY165 and MPY17 strains (Fig. 5).

To confirm this link between Cu reduction and higher Cu uptake, we used the SKY FRE strain in which *FRE1* expression is rescued. The reduction rate for this strain was 2.07 ± 0.32 μmol.l^−1^.min^−1^, which is twice that of SKY34 (Fig. 4). The consequence of this higher reduction rate is that twice as much Cu was taken up by SKY FRE cells (Fig. 5). This indicated that the availability of Cu in its transportable form Cu(I) for the cell is limiting for the uptake of copper by SKY34 cells under normal culture conditions. Under normal culture conditions, the isotopic composition of the SKY FRE strain varies between that of the SKY34 and the wild type DTY165 strains (Fig. 5). We conclude that the greater the ability for the cell to reduce Cu, the stronger the enrichment in light Cu.

Finally, the Δ^65^Cu_cell_ of DTY165 reached its minimal value (−0.85‰/10^8^cells) with small oscillations with a maximum oscillation rate of 7.43 10^−3^‰/10^8 ^cells/min. The maximum oscillation rate for SKY34 is similar: 7.60 10^−3^‰/10^8 ^cells/min. In the strain without high-affinity importers (MPY17), the maximum oscillation rate is slower (3.09 10^−3^‰/10^8 ^cells/min).

## Discussion

### Copper fractionation by Cu importers during transport into the cell is modulated by the cell reductive ability

**Figure 1:**
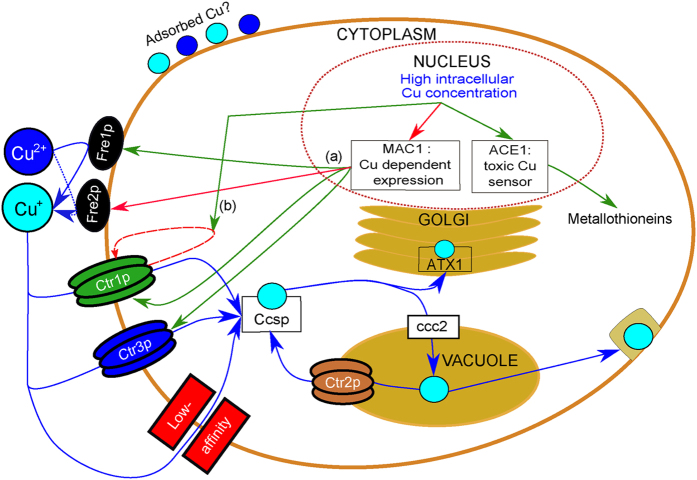
Copper pathways in *Saccharomyces cerevisiae*. Copper fluxes are represented by blue arrows. Regulatory loops are indicated in red (activation) and green (repression). Monovalent and divalent Cu are represented by dark blue and light blue circles, respectively. The first step of Cu import is the reduction of Cu(II) to Cu(I) by the *Fre* reductases, followed by the import through either high-affinity transporters represented in green (*Ctr1p*) and blue (*Ctr3p*) or low-affinity transporters such as *Fet* proteins (red). Copper is subsequently transported in the different compartments of the cell cytoplasm by different protein chaperones such as *Ccsp, ATX1* or *ccc2*. Copper transport is regulated by *MAC1* which senses high intracellular Cu concentrations and down regulates the expression of the high-affinity transporters *CTR1* and *CTR3* and of the reductase *FRE1*, while it activates the expression of the *FRE2* reductase^3^,^14^,^15^,^16^,^17^,^18^,^21^,^22^,^23^,^24^,^25^,^52^.

In this study, we used *S. cerevisiae* as a model system to explore the origin of copper fractionation in cells, notably because its Cu transport mechanisms are similar to those of human cells (Fig. 1)^3^. *S. cerevisiae* is a versatile genetic model allowing the impact of genetic mutations on cell functions to be tested. The present study demonstrates a preferential ^63^Cu uptake by yeast, consistent with observations on bacteria and plants^29^,^35^. To trace the preference for one of the two Cu isotopes, we used the Δ^65^Cu_cell_ notation (see Results for details). If Δ^65^Cu_cell_ is negative, there is a preferential uptake of ^63^Cu, *i.e*. an enrichment in the light Cu isotope. Under steady-state conditions, there is an enrichment in light Cu that after 240 min reaches Δ^65^Cu_cell_ = −0.85 ± 0.06‰/10^8^ cells. Three mechanisms can fractionate copper isotopes in cells: adsorption on cell surface, reduction of divalent to monovalent ions, and transport through the cell membrane (Fig. 1). Adsorption of Cu on organic or mineral surfaces is known to induce isotopic fractionation depending on both the type of surface and the environmental conditions^29^,^36^,^37^,^38^. The present results clearly show that adsorption on the surface of the cells is very limited (Fig. 3), which rules out adsorption as a mechanism generating Cu fractionation in *S. cerevisiae*.

**Figure 2:**
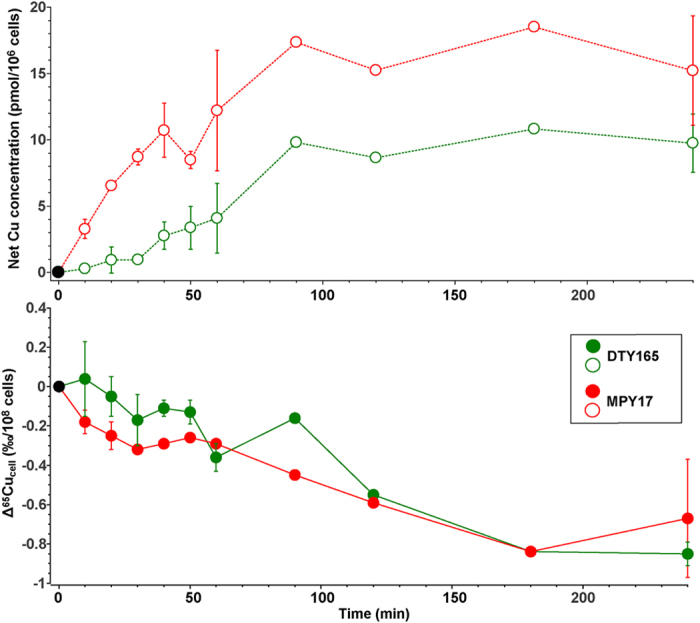
Net variations of the Cu concentration and isotopic composition in *S. cerevisiae* for the wild-type (green) and the Δctr1Δctr3 double-knockout strain (red). Upper panel: variations of the Cu concentration over 240 min. during uptake experiments. Values are reported as the difference Cu(t) − Cu(t = 0) normalized to 10^8^ cells (see text for details). Lower panel: variations of the Cu isotopic composition (reported as Δ^65^Cu_cell_ = δ^65^Cu(t) − δ^65^Cu(t = 0), see text for details) normalized to 10^8^ cells. The error bars are the standard deviation between independent experiments (see Table S1 for details).

**Figure 3:**
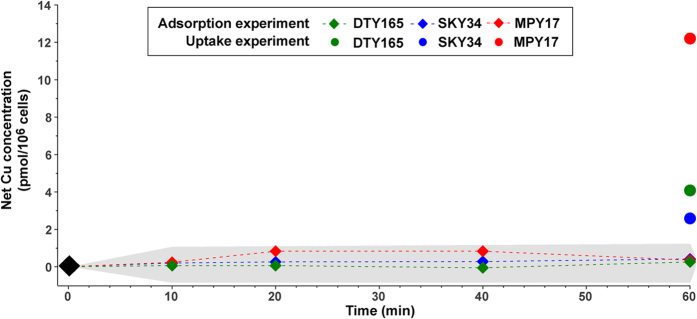
Cu adsorption on *S. cerevisiae* cells (diamonds) over 60 min. Cell contents (circles) at 60 min in uptake experiments are indicated for comparison. The grey range represents the analytical uncertainty on the measurement.

In our experiments, the impact of cellular export, notably exocytosis (Fig. 1), can be safely neglected since in *S. cerevisiae* Cu homeostasis is mainly regulated at the level of the import^3^. Indeed, after ca. 120–180 min (depending on the strain) and up to 240 min, Cu concentrations stay relatively stable for all the studied *S. cerevisiae* strains. Cu isotopic compositions do also stay relatively stable after ca. 120–180 min (Figs 2 and 5). Therefore, we assume that, after 180 min, inward and outward Cu fluxes across the *S. cerevisiae* cell membrane are very small compared to what occurred during the Cu uptake phase, *i.e*. before 120–180 min, depending on the strain. As a consequence, the Cu isotopic fractionation due to Cu uptake by *S. cerevisiae* is likely to be very small compared to what is happening during the uptake phase. Thus, we assume that the changes in Cu isotopic fractionation due to Cu uptake can be neglected once the plateau is reached. In addition, strains compared in this study have similar genetic backgrounds for the export. Thus, any potential effect of export on the intracellular Cu isotopic composition should have been similar for all strains. Similarly, after 180 min, the Cu isotopic fractionation due to Cu uptake by *S. cerevisiae* was likely to be very small compared to what was happening in the phase during which the intracellular concentration increased, *i.e*. before 120–180 min. This is indeed what we observed (Figs 2 and 5).

It is known from both experimental studies and *ab initio* calculations that changes in the oxidation state of Cu can lead to fractionation^39^,^40^,^41^,^42^. To estimate the role of the reductase on Cu isotopic fractionation in *S. cerevisiae* using biological approaches, we used a Cu reductase deficient mutant (SKY34). In this strain, Cu uptake was similar to the wild type but there was virtually no Cu isotopic fractionation (Δ^65^Cu_SKY34_ = −0.02 ± 0.07‰/10^8^cells). To distinguish the role of reductase from the role of importers, the copper-reductase deficient SKY34 strain was fed solely with monovalent copper using ascorbic acid to ensure full reduction. In this case, Cu isotope fractionation is similar to that of the wild type strain (Δ^65^Cu_SKY34, ascorbic acid_ = −0.90 ± 0.02‰/10^8^cells, Fig. 5). In the experiments with ascorbic acid, there is no Cu(I) derived from reductase activity. Thus, it is reasonable to assume that Cu fractionation in *S. cerevisiae* is due to the passage of Cu through transmembrane importers.

**Figure 4:**
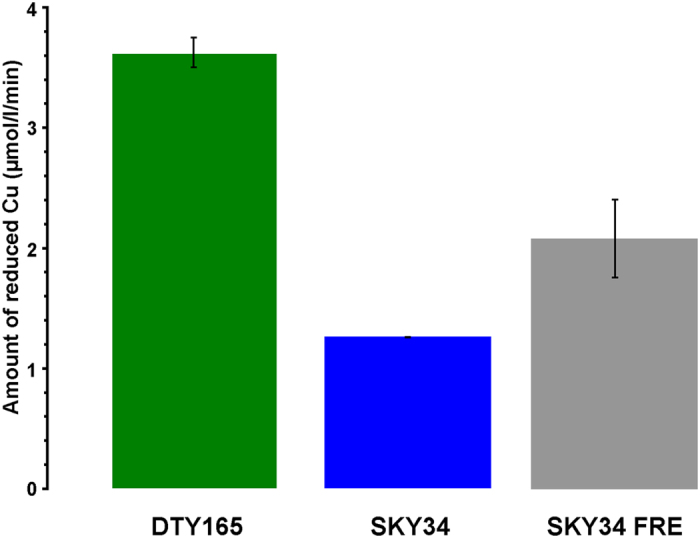
Copper reductase activity in DTY165, SKY34, and SKY FRE. Amount of reduced Cu for the wild type strain (DTY165), the Δctr1Δmac1 knockout strain (SKY34), and the SKY34 strain where *FRE1* is under the control of ADH promoter (SKY FRE). All experiments were duplicated. Error bars are the 2σ on the measurements. For SKY 34, error bar is lower than 0.05 μmol/L/min.

**Figure 5:**
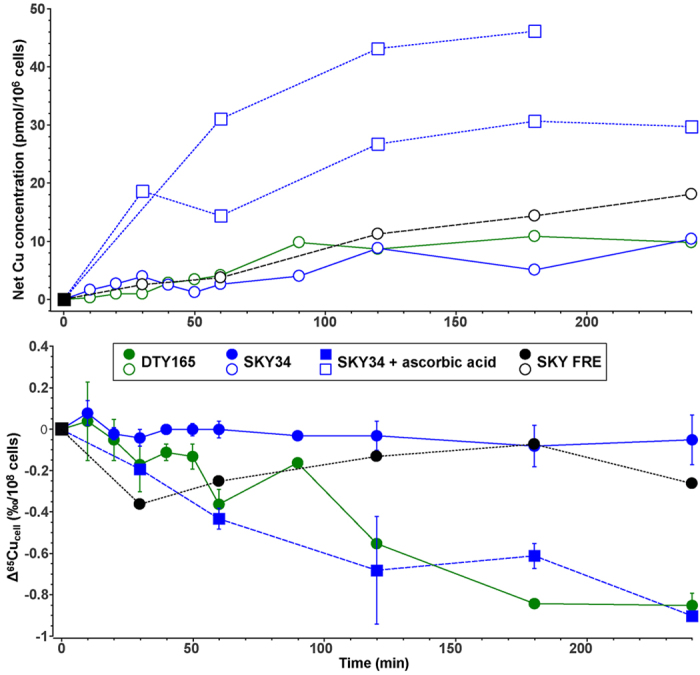
Net variations of the Cu concentration and isotopic composition in *S. cerevisiae* for the wild-type (DTY165, green), the Δctr1Δmac1 double-knockout strain (SKY34, blue), and the Δctr1Δmac1 strain where FRE1 is under the promoter of ADH (SKY FRE, black). Upper panel: variations of the Cu concentration (see Fig. 2 caption for details) during uptake experiments in absence (circles) or presence of ascorbic acid (squares). Lower panel: Variations of the Cu isotopic composition (see Fig. 2 caption for details) during uptake experiments in absence (circles) or presence of ascorbic acid (squares). The error bars are the standard deviation between independent experiments (see Table S1 for details).

To explain the absence of fractionation for SKY34 cultivated under normal growth conditions, we hypothesize that the availability of transportable Cu(I) for the cell is limiting because the reductase activity is twice lower compared to the wild type. As a consequence, all Cu(I) produced by the reductase activity is transported inside the cell by the importers, which does not allow for fractionation of copper isotopes at that step. This hypothesis is corroborated by the fact that when copper is directly provided in excess as monovalent ions to this strain, *i.e*. in the ascorbic acid uptake experiments, isotopic fractionation does occur and reaches that of the wild type (Fig. 5). When the ability of SKY34 to reduce Cu is enhanced, *i.e*. in the SKY FRE strain (Fig. 4), the Cu(I) pool increases which permits the fractionation by importers. We indeed observe a decrease of the Δ^65^Cu_cell_ for the SKY FRE strain which falls between the values of the wild type and SKY 34 (Fig. 5). Thus, the ability of cells to reduce Cu controls the quantity of available Cu(I) which in turn modulates the amplitude of the isotopic fractionation. This is the first experimental demonstration of a direct link between the activity of transporters and isotopic fractionation.

### Copper isotopic compositions fluctuate with time

Copper transport in *S. cerevisiae* is tightly regulated. It has been demonstrated that high intracellular Cu concentrations lead to a rapid decrease of the *CTR3* gene expression^43^ and to a rapid decrease of the membrane *CTR1* content, which in turn leads to the reduction of copper entry^24^,^25^. The extremely rapid and efficient degradation of the transporter proteins leads to a drastic depletion at the membrane, which is compensated by fine-tuning of the expression of the transporter genes. These regulations generate specific and very reproducible oscillations in transcription levels in the first ca. 90 min^43^. During our monitoring of *S. cerevisiae*, we observed oscillations of the isotopic composition of copper in strains expressing high-affinity transporters, *i.e*. the wild-type DTY165 and SKY34 strains (Fig. 5). A possible explanation for these oscillations may be that they occur in response to the oscillations in the transcriptional regulation of the high-affinity transporters and may thus result from the fine-tuning of Cu transport. Demonstration of a direct or indirect connection between copper uptake regulation and the oscillations of the isotopic phenotype of yeast would require additional work using a different experimental setting, and is beyond the scope of this study.

### Affinity interactions with the reductases and transporters generate Cu fractionation

Cu(II) present in the growth medium, either free or more likely bound to various ligands^5^, is reduced by the Fre1p and Fre2p proteins^14^,^15^. However, there is no current understanding of how Cu(I) is maintained in its reduced form prior to its uptake by the high-affinity copper importers *CTR1* and *CTR3*^44^. There could be a direct interaction between the reductases and the transporters or intermediate ligands that bind Cu(I). Extracellular Cu(I) is bound to the amino acids present on the N-terminal domain of the high-affinity Cu importers: methionine (Met), histidine (His), and cysteine (Cys)^45^. *CTR1* notably has methionine-rich sites (Mets motifs) that are thought to bind Cu(I) prior to its transport across the membrane^46^. *CTR3* lacks these Met-motifs on its extracellular side but has an abundance of cysteine residues^47^ that could bind extracellular Cu(I). The precise transmembrane transport mechanism is yet not fully understood. It has been proposed that exchanges between several binding sites allow Cu(I) to move through the membrane to the amino acid residues located at the C terminus of the *Ctr* proteins where it is presented to cytosolic Cu chaperones^46^,^47^,^48^. The intracellular C domain of *CTR1 and CTR3* contains Cys/His motifs that can bind Cu(I)^49^. *Ab initio* calculations give a theoretical prediction of the isotopic fractionation occurring between metal ligands. Several Cu(II) ligands have already been studied^39^,^41^. Similarly, we can determine an isotopic fractionation between the amino acid complexes that bind extracellular Cu(I) and the cytosolic Cys/His motifs of the importers. We calculated the values of the isotopologue partition functions ratio lnβ for the various Cu(I)-amino acid complexes (Supplementary Table S3) to calculate the theoretical fractionation induced by the transfer of Cu(I) from Met (*CTR1*) and Cys (*CTR3*) to His or Cys ligands. Histidine has two coordination sites that we have named His(1) and His(2), with His(1) having the largest lnβ for Cu(I). At the temperature of our experiments (298 K), the calculations predict a ^63^Cu enrichment due to the transfer of Cu(I) through the transporters for exchanges between Met and His(1) for *CTR1* (δ^65^Cu = −0.78‰), and Cys and His(1) for *CTR3* (δ^65^Cu = −0.73‰). While *ab initio* calculations represent a simplified approach to the complexity of the biological mechanisms and the match between the experimental results and *ab initio* calculations is not perfect, the amplitude of the fractionation predicted by *ab initio* calculation are, to the first order, in agreement with the experimental results presented in this study. Thus, the cellular enrichment in ^63^Cu in the wild type strain DTY165, in the SKY34 mutant with ascorbic acid, and in the SKY FRE mutant could be explained by the transport of the extracellular Cu(I) bound to the sulfur-rich amino acids methionine and cysteine to the cytosolic histidine-containing motifs of the high-affinity *CTR1* and *CTR3* transporters. Interestingly, experiments performed with MPY17 which lacks the high-affinity copper transporters show that Cu is fractionated to an extent only slightly lower than that of the wild type (Fig. 2). Although it is difficult to know which low-affinity transporters are responsible for Cu transport in strain MPY17, our observations and *ab initio* calculation results suggest that several steps of chelation of copper might also be involved in the transmembrane transfer of Cu by these low-affinity transporters.

### Using Cu isotopic composition as a prognostic tool for cancer

In this study, we show that a decrease in the overall capacity of the cell to reduce Cu via an impaired transcription of the reductases leads to an increase of the SKY34 cells δ^65^Cu of *ca*. 0.9‰ compared to that of the wild type without modification of the intracellular Cu concentration (Fig. 5). This situation is close to what is observed in hepatocellular carcinoma (HCC) patients, for which δ^65^Cu is higher in the tumor relative to the peritumor, despite no significant differences in Cu concentration^9^. Such an enrichment in the tumor is mirrored by lower δ^65^Cu in the serum of HCC patients compared to healthy donors. In human cells, Cu is reduced by *FRE* homologs, the STEAP proteins^50^. Intriguingly, in HCC patients, immunohistochemical evidence shows that the level of STEAP3 protein is lower in the tumor mass compared to the surrounding tissue^13^. Moreover, STEAP3 expression has been shown to be a good prognostic marker of the transition from cirrhosis to HCC^12^,^13^. We hypothesize that the mechanism leading to Cu isotope fractionation observed in HCC is similar to the mechanism inferred for *S. cerevisiae*: a decrease in the ability to reduce Cu via a lower expression of STEAP3 protein leads to higher cellular δ^65^Cu values compared to the surrounding healthy tissues. It has also been shown that the increase of the δ^65^Cu values in the tumor is correlated with a decrease of the δ^65^Cu values in red blood cells^9^. Thus, monitoring the δ^65^Cu in red blood cells might constitute a non-invasive prognostic test to quickly detect the transition from liver cirrhosis to carcinoma.

## Conclusions

In the present study, we have demonstrated that the main mechanism responsible for the enrichment in light Cu isotope in the model organism *S. cerevisiae* is the import of Cu via high- (Ctr1p and Ctr3p) and low-affinity transporters. We have also shown that the ability of the cell to reduce Cu modulates the amplitude of the fractionation. Copper adsorption on the membrane does not play a significant role. Based on *ab initio* calculation results, we propose that Cu fractionation may be due to the binding of Cu(I) with the sulfur-rich amino acids of the metallic center of the transporter, cysteine and methionine and its subsequent transport to the cytosol. Because of the high conservation of Cu transport in eukaryotic cells, our observations can be extended to Cu homeostasis in human cells. During the transition from cirrhosis to hepatocellular carcinoma, the expression of copper reductases is decreased and an enrichment in heavy Cu isotopes in the tumors is observed. Our study suggests that these two observations may be functionally connected and thus δ^65^Cu could be a good tool to trace changes in Cu fluxes in liver cancer patients.

## Additional Information

**How to cite this article:** Cadiou, J.-L. *et al*. Copper transporters are responsible for copper isotopic fractionation in eukaryotic cells. *Sci. Rep.* **7**, 44533; doi: 10.1038/srep44533 (2017).

**Publisher's note:** Springer Nature remains neutral with regard to jurisdictional claims in published maps and institutional affiliations.

## Supplementary Material

Supplementary Information

## Figures and Tables

**Table 1:** Genotype of the four studied *S. cerevisiae* strains.

Strain	Cu importers		Cu transcription factor	Cu reductases	activity	Genotype	Reference
	Ctr1	Ctr3	MAC1	FRE 1 promoter			
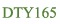	(+)	(+)	(+)	wt	+++	MATα ura3-52 his6 leu2-3,-112 his3-∆200 trp1-901 lys2-801 suc2-∆	51
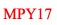	(−)	(−)	(+)	wt	+++	MATα ctr1::ura3::Kanr ctr3::TRP1 his3 lys2-801 CUP1^r^	47
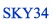	(−)	(+)	(−)	wt	+	MATα gal1 trp1-1 his3 ade8 CUP1^r^ ctr1::TRP1 mac1	47
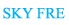	(−)	(+)	(−)	ADH	++	MATα gal1 trp1-1 his3 ade8 CUP1^r^ ctr1::TRP1 mac1 pFRE1∆::Kanr-pADH	This study

(−) knockout, (+) expressed gene.

The relative Cu reductase activity is given by the number of ‘+’ in the 6^th^ column (“activity”). Colors used in this table for each strain are the ones used in the figures.
