# Supplementary material for: Copper transporters are responsible for copper isotopic fractionation in eukaryotic cells
Source: Sci Rep. 2017 Mar 17;7:44533. doi: 10.1038/srep44533 (PMC5356015; doi:10.1038/srep44533)
Supplement: Supplementary Information [file srep44533-s1.pdf]

Supplementary Information

Copper transporters are responsible for copper isotopic fractionation in eukaryotic cells

Jean-Loup Cadiou[1], Sylvain Pichat[1,*], Victor P. Bondanese[1], Alexandre Soulard[2], Toshiyuki Fujii[3], Francis Albarède[1], Philippe Oger[1,4,*]

[1] Univ Lyon, ENS de Lyon, Université Claude Bernard Lyon 1, CNRS, UMR 5276, Lyon, France
[2] Univ Lyon, Université Claude Bernard Lyon 1, INSA-Lyon, CNRS, UMR5240, Villeurbanne, France
[3] Division of Sustainable Energy and Environmental Engineering, Graduate School of Engineering, Osaka University, Osaka, Japan
[4] Univ Lyon, INSA-Lyon, Université Claude Bernard Lyon 1, CNRS, UMR5240, Villeurbanne, France

* Corresponding authors
e-mail: sylvain.pichat@ens-lyon1.fr and philippe.oger@insa-lyon.fr

The supplementary information includes:

Table S1. Calculated average relative Cu concentrations and Cu isotopic compositions

Table S2. Isotopic composition measurement of the same *S. cerevisiae* sample aliquot during four runs over the time course of 3 months

Fig. S1. Evolution of Cu concentrations at a given time over Cu initial concentration and isotopic composition in the growth medium as a function of time.

Table S3. Logarithm of the reduced partition function, lnβ (‰), for the pair ^{65}Cu–^{63}Cu of Cu(I)–amino acid complexes with a description of the computational methods.

Table S1. Calculated net Cu concentrations and Cu isotopic compositions (see text as well as (1) and (2) below for details).
NA: not applicable

Strain	Time step (min)	Number of replicate experiments	Average net Cu concentration (ppm/10^6 cells) (1)	S.D. between replicate experiments	Average $\Delta^{65}Cu_{cell}(t)$ (‰/10^8 cells) (2)	S.D. between replicate experiments	For each replicate experiment: Number of replicate measurements (3)	For each replicate experiment: S.D. between replicate measurements (4)
DTY165	0	3	0		0		1 / 4 / 1	NA / 0.01/ NA
	10	2	0.27	*0.23*	0.04	*0.19*	4 / 3	0.06 / 0.03
	20	2	0.93	*0.98*	-0.05	*0.10*	2 / 3	0.04 / 0.02
	30	2	0.95	*0.27*	-0.17	*0.13*	4 / 3	0.04 / 0.00
	40	2	2.77	*1.03*	-0.11	*0.04*	4 / 3	0.06 / 0.02
	50	2	3.37	*1.62*	-0.13	*0.06*	4 / 2	0.04 / 0.00
	60	2	4.09	*2.62*	-0.36	*0.07*	4 / 3	0.01 / 0.05
	90	1	9.80	*NA*	-0.16	*NA*	1	NA
	120	1	8.66	*NA*	-0.55	*NA*	1	NA
	180	1	10.81	*NA*	-0.84	*NA*	1	NA
	240	3	8.97	*2.05*	-0.85	*0.06*	1 / 2 / 3	NA / 0.04 / 0.01
MPY17	0	3	0		0		1 / 8	NA / 0.02
	10	2	3.27	*0.71*	-0.18	*0.09*	1 / 5	NA / 0.03
	20	2	6.54	*0.32*	-0.25	*0.10*	2 / 5	0.11 / 0.04
	30	2	8.70	*0.59*	-0.32	*0.02*	2 / 4	0.04 / 0.02
	40	2	10.72	*2.05*	-0.29	*0.01*	4 / 5	0.01 / 0.03
	50	2	8.49	*0.65*	-0.26	*0.02*	4 / 5	0.08 / 0.05
	60	2	12.21	*4.55*	-0.29	*0.02*	4 / 4	0.07 / 0.02
	90	1	17.35	*NA*	-0.45	*NA*	2	0.00
	120	1	15.23	*NA*	-0.59	*NA*	2	0.00
	180	1	18.52	*NA*	-0.84	*NA*	2	0.02
	240	2	15.22	*4.13*	-0.67	*0.30*	2 / 6	0.02 / 0.02

Strain	Time step (min)	Number of replicate experiments	Average net Cu concentration (ppm/10^6 cells) (1)	S.D. between replicate experiments	Average $\Delta^{65}Cu_{cell}(t)$ (‰/10^8 cells) (2)	S.D. between replicate experiments	For each replicate experiment: Number of replicate measurements (3)	For each replicate experiment: S.D. between replicate measurements (4)
SKY34	0	5	0		0		1 / 4 / 2 / 2 / 1	NA / 0.05 / 0.01 / 0.12 / NA
	10	2	1.61	*1.29*	0.08	*0.06*	2 / 3	0.02 / 0.05
	20	2	2.68	*2.09*	-0.02	*0.03*	2 / 4 / 3	0.06 / 0.08
	30	3	4.98	*2.60*	-0.04	*0.04*	1 / 3	NA / 0.06 / 0.07
	40	2	2.49	*0.32*	0.00	*0.01*	4 / 3	0.1 / 0.03
	50	2	1.16	*1.36*	0.00	*0.03*	1 / 3	NA / 0.02
	60	3	5.52	*5.08*	0.00	*0.04*	1 / 4 / 4 / 3	NA / 0.01 / 0.05 / 0.01
	90	1	3.96	*NA*	-0.03	*NA*	1	NA
	120	2	8.89	*0.14*	-0.03	*0.07*	2 / 3	0.03 / 0.06
	180	2	7.50	*3.45*	-0.08	*0.10*	2 / 3	0.00 / 0.00
	240	5	9.69	*3.02*	-0.02	*0.07*	2 / 2 / 2 / 2 / 3	0.01 / 0.03 / 0.03 / 0.00
SKY34 + ascorbic acid	0	3	0		0		2 / 4 / 3	0.01 / 0.00 / 0.06
	30	1	19.72	*NA*	-0.19	*NA*	3	0.03
	60	2	22.70	*11.83*	-0.43	*0.05*	2 / 3	0.02 / 0.03
	120	3	27.67	*15.00*	-0.68	*0.26*	4 / 2 / 3	0.01 / 0.03 / 0.00
	180	2	36.95	*8.91*	-0.61	*0.06*	4 / 2	0.01 / 0.06
	240	2	22.68	*9.98*	-0.90	*0.02*	4 / 3	0.02 / 0.03
SKY FRE	0	1	0		0		3	0.01
	30	1	2.50	*NA*	-0.36	*NA*	3	0.02
	60	1	3.75	*NA*	-0.25	*NA*	3	0.03
	120	1	11.25	*NA*	-0.13	*NA*	3	0.03
	180	1	14.36	*NA*	-0.07	*NA*	3	0.03
	240	1	18.06	*NA*	-0.26	*NA*	3	0.02

(1) The net Cu concentration reported here is: Cu(t) - Cu(t=0), normalized to 10^6 cells (see Materials and methods for details).

(2) $\Delta^{65}Cu_{cell}$ (t) = Δ^{65}Cu (t) - Δ^{65}Cu (0) with Δ^{65}Cu (t) = $\delta^{65}Cu_{cell}$ (t) - $\delta^{65}Cu_{medium}$.
Thus, $\Delta^{65}Cu_{cell}$ (t) = ($\delta^{65}Cu_{cell}$ (t) - $\delta^{65}Cu_{medium}$) - ($\delta^{65}Cu_{cell}$ (0) - $\delta^{65}Cu_{medium}$) = $\delta^{65}Cu_{cell}$ (t) - $\delta^{65}Cu_{cell}$ (0).
The $\Delta^{65}Cu_{cell}$ (t) value was then normalized to 10^8 cells (see Materials and methods for details).

For example, for DTY165, at t = 240 min, in the third independent experiment (3rd replicate experiment) the $\delta^{65}Cu_{DTY165}$ (240) was measured 3 times, *i.e.* 3 replicate measurements, and the average value was $\delta^{65}Cu_{DTY165}$(240) = -1.84 ± 0.01‰ (1σ). The $\delta^{65}Cu_{DTY165}$(0) was measured at -1.22‰, thus $\Delta^{65}Cu_{DTY165}$(240) = -0.62‰. The number of cells in the 240 min sample was 0.68×10^8 cells. Thus, the normalized value of the $\Delta^{65}Cu_{DTY165}$(240) for the third experiment was calculated to be -0.91‰/10^8 cells.

(3) number of replicate measurements of the Cu isotopic composition for each culture replicate experiment, e.g. “4 / 3” means that 4 replicate measurements were made on the first replicate experiment and 3 on the second replicate experiment.

(4) standard deviation (S.D.) between the replicate measurements made for each replicate experiment.

Table S2. Isotopic composition measurement of the same *S. cerevisiae* sample aliquot during four runs over the time course of 3 months.

**Sample**	**Date**	**δ^{65}Cu (‰)**
MPY 17, t=240min	31/04/2015	-1.46
		-1.49
		-1.46
	04/07/2015	-1.46
	14/07/2015	-1.51
	31/07/2015	-1.47
		-1.47
		-1.46
	**Average**	**-1.47 ± 0.04** (2σ)

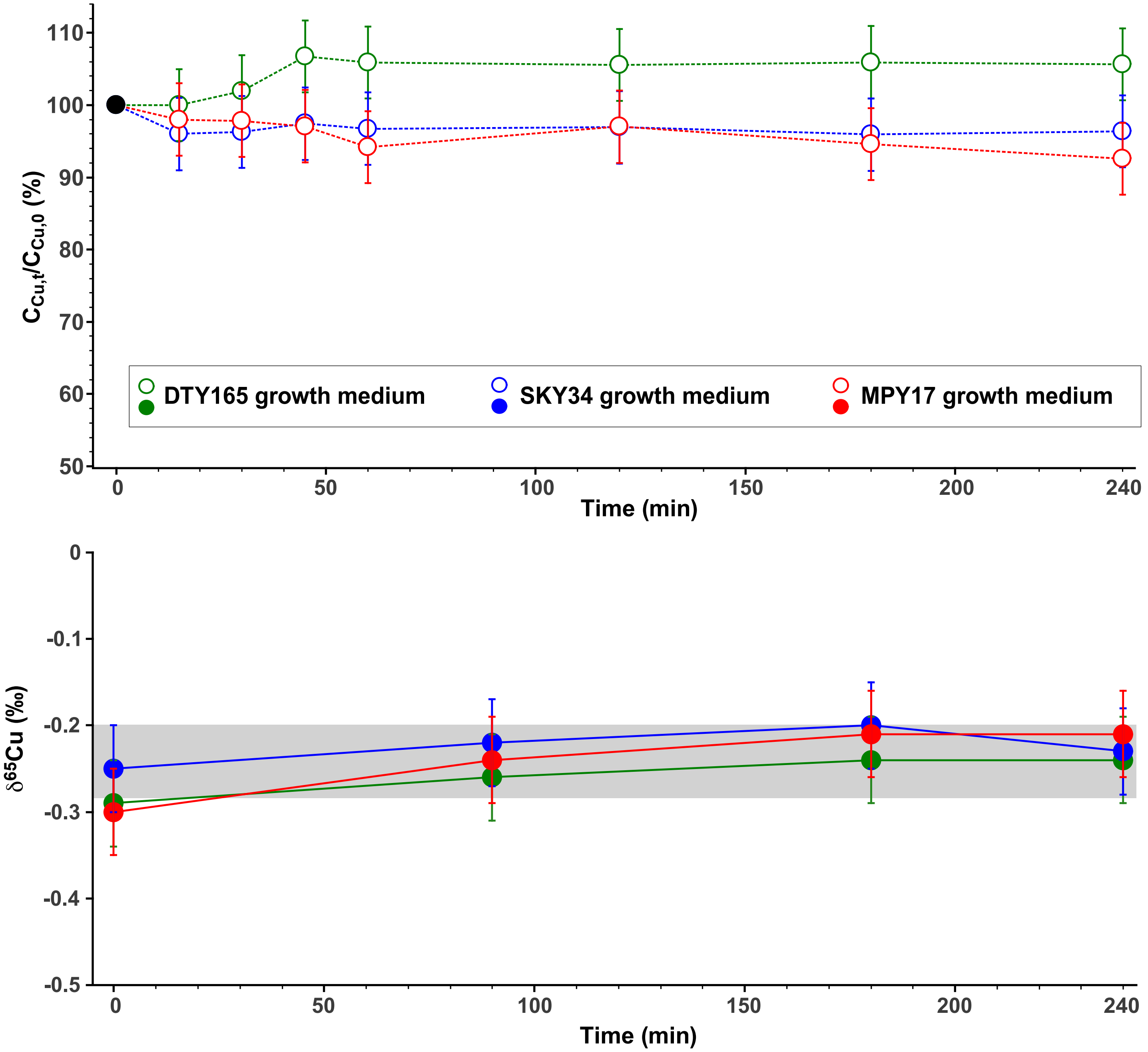

Figure S1: Evolution of Cu concentrations at a given time over Cu initial concentration (upper panel) and isotopic composition (lower panel) in the growth medium as a function of time. The grey area represents the analytical uncertainty of the measures (2σ = 0.1‰).

Table S3. Logarithm of the reduced partition function, lnβ (‰), for the pair ^{65}Cu–^{63}Cu of Cu(I)–amino acid complexes. Computational methods are given below.

		Temperature (K)				
		273	298	310	323	373
$Cu^I(Met)(H_2O)^+$		3.101	2.628	2.438	2.254	1.710
$Cu^I(Cys)(H_2O)^+$		3.157	2.677	2.483	2.296	1.742
$Cu^I(His)(H_2O)^+$	His (1)	4.013	3.408	3.165	2.930	2.230
$Cu^I(His)(H_2O)^+$	His (2)	2.474	2.090	1.937	1.789	1.353

Computational methods

Orbital geometries and vibrational frequencies of aqueous Cu(I) species were computed using the density functional theory (DFT) as implemented by the Gaussian09 code[1-2]. The DFT method employed here is a hybrid density functional consisting of Becke's three-parameter non-local hybrid exchange potential (B3)[3] with Lee *et al.* (LYP)[4] non-local functionals. Using the 6-311+G(d,p) basis set or higher is recommended for calculating the Cu complexes by de Bruin *et al.*[5]. The 6-311+G(d,p) basis set, which is an all-electron basis set, was therefore chosen for H, C, N, O, S, and Cu. Molecules were modeled without any forced symmetry. An "ultrafine" numerical integration grid was used and the SCF (self-consistent field) convergence criterion was set to 10^{-8}.

The isotope enrichment factor due to intramolecular vibrations can then be evaluated from the reduced partition function ratio[6] (*s/s'*)*f*, also noted β,

$$ln\frac{s}{s'}f = \sum\left[ln\,b(u_i') - ln\,b(u_i)\right] \quad (1)$$

where

$$ln\,b(u_i) = -ln\,u_i + \frac{u_i}{2} + ln\left(1 - e^{-u_i}\right) \quad (2)$$

and

$$u_i = \frac{h\nu_i}{kT} \quad (3)$$

in which ν stands for vibrational frequency, *s* for the symmetry number of the Cu compound, *h* the Plank constant, *k* the Boltzmann constant, and *T* the absolute temperature. The subscript *i* denotes the ith mode of molecular vibration, and primed variables refer to the light isotopologue. The isotope enrichment factor due to molecular vibrations can be evaluated from the frequencies (ν) summed over all the different modes.

References

1. Dennington, R., Keith, T. & Millam, J. GaussView, *Version 5.0.9*. Semichem Inc., Shawnee Mission KS (2009).

2. Frisch, M. J. *et al.* Gaussian 09, *Revision E.01*, Gaussian, Inc., Wallingford CT (2009).

3. Becke, A. D. Density-functional thermochemistry. III. The role of exact exchange. *J. Chem. Phys.* **98**, 5648-5652, doi:doi:http://dx.doi.org/10.1063/1.464913 (1993).

4. Lee, C., Yang, W. & Parr, R. G. Development of the Colle-Salvetti correlation-energy formula into a functional of the electron density. *Phys. Rev. B* **37**, 785-789 (1988).

5. de Bruin, T. J. M., Marcelis, A. T. M., Zuilhof, H. & Sudholter, E. J. R. Geometry and electronic structure of bis-(glycinato)-Cu $^{II}\cdot 2H_2O$ complexes as studied by density functional B3LYP computations. *Phys. Chem. Chem. Phys.* **1**, 4157-4163, doi:10.1039/a903018b (1999).

6. Bigeleisen, J. & Mayer, M. G. Calculation of Equilibrium Constants for Isotopic Exchange Reactions. *J. Chem. Phys.* **15**, 261-267, doi:doi:http://dx.doi.org/10.1063/1.1746492 (1947).
